# Strategic Approach, Advances, and Challenges in the Development and Application of the SIT for Area-Wide Control of *Aedes albopictus* Mosquitoes in Reunion Island

**DOI:** 10.3390/insects11110770

**Published:** 2020-11-07

**Authors:** Louis Clément Gouagna, David Damiens, Clélia F. Oliva, Sébastien Boyer, Gilbert Le Goff, Cécile Brengues, Jean-Sébastien Dehecq, Jocelyn Raude, Frédéric Simard, Didier Fontenille

**Affiliations:** 1 MIVEGEC, IRD, CNRS, Université Montpellier, CEDEX 5, 34394 Montpellier, France; david.damiens@ird.fr (D.D.); clelia.oliva@ctifl.fr (C.F.O.); gilbert.legoff@ird.fr (G.L.G.); cecile.brengues@ird.fr (C.B.); frederic.simard@ird.fr (F.S.); didier.fontenille@ird.fr (D.F.); 2 Medical and Veterinary Entomology Unit, Institut Pasteur du Cambodge, Phnom Penh 12201, Cambodia; sboyer@pasteur-kh.org; 3 ARS—Délégation Départementale de la Haute-Garonne, Pôle de Prévention et Gestion des Alertes Sanitaires, CEDEX 2, 31050 Toulouse, France; jean-sebastien.dehecq@ars.sante.fr; 4 EHESP, School of Public Health, UMR “Emergence des Pathologies Virales”, Université Aix-Marseille, IRD190, INSERM1207, 35043 Rennes, France; jocelyn.raude@ehesp.fr

**Keywords:** sterile insect technique, irradiation, feasibility study, *Aedes albopictus*

## Abstract

**Simple Summary:**

*Aedes albopictus* is a well-established competent arbovirus vector in Reunion Island, a French overseas territory in the Indian Ocean, occurring in a range of natural to urban environments where it represents a major threat to public health. Following the 2006 Chikungunya outbreak and periodic occurrence of dengue epidemics, the sterile insect technique (SIT) emerged as the most environment-friendly option for integration with the current vector control strategy that relies mainly on the elimination of breeding sites and insecticide applications. This paper describes the trajectory that has been followed in assessing the feasibility of SIT against *Ae. albopictus* in Reunion Island, and reviews some of the main achievements since 2009. These include essential scientific information so far obtained on the biology and ecology of *Ae. albopictus*, and the development of the requisite technological capabilities for the production and release of sexually competitive sterile males. Furthermore, it also draws attention to the strategies established to streamline the decision-making process, including an awareness campaign to enhance public understanding, efforts to secure public acceptance and regulatory validation of SIT pilot testing for small-scale suppression of wild *Ae. albopictus* in selected urban sites on the island.

**Abstract:**

The global expansion of *Aedes albopictus,* together with the absence of specific treatment and vaccines for most of the arboviruses it transmits, has stimulated the development of more sustainable and ecologically acceptable methods for control of disease transmission through the suppression of natural vector populations. The sterile insect technique (SIT) is rapidly evolving as an additional tool for mosquito control, offering an efficient and more environment-friendly alternative to the use of insecticides. Following the devastating chikungunya outbreak, which affected 38% of the population on Reunion Island (a French overseas territory in the southwest of the Indian Ocean), there has been strong interest and political will to develop effective alternatives to the existing vector control strategies. Over the past 10 years, the French Research and Development Institute (IRD) has established an SIT feasibility program against *Ae. albopictus* on Reunion Island in collaboration with national and international partners. This program aimed to determine whether the SIT based on the release of radiation-sterilized males is scientifically and technically feasible, and socially acceptable as part of a control strategy targeting the local *Ae. albopictus* population. This paper provides a review of a multi-year and a particularly broad scoping process of establishing the scientific and technological feasibility of the SIT against *Ae. albopictus* on Reunion Island. It also draws attention to some prerequisites of the decision-making process, through awareness campaigns to enhance public understanding and support, social adoption, and regulatory validation of the SIT pilot tests.

## 1. Introduction

The invasion of *Aedes* (*Stegomyia*) mosquito species in many countries worldwide is strongly associated with progressive dissemination and persistence of various vector-borne diseases in human populations, such as dengue, chikungunya, and Zika [1,2]. More than 100 countries, encompassing almost half of the world’s population, are considered to be at risk for these arboviruses [3] and their impact remains a major challenge to health-care authorities. Already faced with the devastating epidemic of chikungunya in 2005–2006 that affected more than 30% of the population [4,5], Reunion Island has been confronted for several years (from 2004 to 2020) with a persistent epidemic of dengue fever. The 2020 dengue epidemic in La Reunion involved mostly autochthonous cases of serotype DENV-2, followed by DENV-1 and DENV-3, with 2–3 serotypes being frequently circulated simultaneously [6].

From an entomological point of view, Reunion Island hosts about 12 mosquito species [7], including *Aedes aegypti* and *Aedes albopictus,* which are both considered invasive in la Reunion. The latter is considered the predominant mosquito species and is the main vector of dengue and of chikungunya in the outbreaks of 2005–2006 [8]. The evidence that *Ae. albopictus* is a competent vector of dengue and chikungunya in Reunion Island has been shown in several studies [9,10]. The magnitude of the public health risk in the island is due to *Ae. albopictus*’ widespread distribution at high densities in a plethora of artificial and natural breeding sites. It thrives mostly in urban areas where it is the only species responsible for viral transmission, while *Ae. aegypti* is more confined to natural habitats including areas of active dengue transmission. As *Ae. aegypti* is less anthropophilic in La Reunion, its importance in the locally occurring arboviral epidemics is still unknown. The ecological mechanisms underpinning differences in the spatial distribution of *Ae. albopictus* and *Ae. aegypti* on La Reunion are well understood [11,12,13,14]. A study carried out from 2009 to 2011 [15] highlighted the strong adaptation potential of the local *Ae. albopictus* populations, in particular through movement between natural habitats (e.g., ravines) and residential areas.

The French Ministry of Health has defined an area-wide-vector-management strategy combining source reduction (elimination of breeding habitats) in part through the local communities, protection of individuals against mosquito bites with repellents or mosquito nets, stakeholder involvement and the application of larvicides (*Bti*). Generally, the use of insecticides (deltamethrin) against *Aedes* mosquito adults involves daytime peri-domestic treatments or space spraying at night, carried out around biologically confirmed cases, and exceptionally around suspected cases. Although the current strategies have allowed containing the different epidemic waves by reducing the number of new cases to proportions that remain medically manageable, non-compliance of residents to apply control measures compromise the effectiveness of breeding site elimination throughout urban and suburban areas. Moreover, active breeding site elimination has been difficult in view of the high ecological diversity of the affected zones and difficult topography of the island. In addition, the targeted used of insecticide sprays on a localized basis over urban areas, has not been effective to manage vector populations on a larger scale. Although the local populations of *Ae. albopictus* have not developed resistance yet to deltamethrin, this recommended insecticide is classified as a broad spectrum adulticide that is potentially toxic to a wide range of beneficial insects and as a result will have a negative impact on the environment [16,17,18]. The intensive insecticide spraying during the chikungunya and dengue epidemic outbreaks has resulted in growing concerns of the general public with respect to its impact on the environment and human health and the possible development of insecticide-resistant insects.

The occurrence of several waves of epidemics of arboviral diseases on Reunion Island questions the effectiveness and sustainability of the attempts made to manage the vector. This situation has however, created an opportunity to develop and implement alternative or additional vector control tactics that satisfy social, environmental, and economic goals. Over the past decades, several technologies have emerged as a result of scientific advances to strengthen the conventional vector control methods. Among the possible options are: (a) The release of fertile genetically modified mosquitoes (GMM) designed to alter the fitness of wild population [19]; (b) the release of transgenic males (e.g., RIDL OX513A strain) to reduce target populations [20,21]; (c) the incompatible insect technique (IIT) that exploits the cytoplasmic incompatibility induction properties of *Wolbachia* by which wild-type females that successfully mate with Wolbachia-carrying males will produce non-viable eggs [22,23]; and (d) the sterile insect technique (SIT) that is based on the release of insects sterilized by ionizing radiation at the target area to reduce the reproduction of a natural population of the same species [24,25]. The comparative analysis of all of these approaches in terms of their objectives, potential for efficiency and durability, and technical constraints as well as the progress to the field has been described in detail elsewhere [26,27]. The decision-making process for the adoption of any of these technology options (SIT/GMM/IIT approach) for the control of disease-transmitting *Aedes* populations is complex. Regardless of the scientific evidence about a technology’s safety and effectiveness, the critical factors that determine their choice depend on political and social feasibility, which may be context-dependent. With particular reference to France, the use of GMM and IIT for vector and pest control faces a number of challenges, particularly as concerns their social and cultural acceptance and regulatory approval [28]. Consequently, the radiation-based approach was considered as the most feasible and safest solution for our island setting.

The SIT is an autocidal control method widely used to suppress or eradicate populations of some of the major insect pests of agricultural, livestock or human health importance. The SIT relies on the production of large numbers of the target species, sterilization of the males using ionizing radiation, and the sustained and systematic release of large numbers of sterile individuals into the target area [24,25]. The goal is to achieve high frequency of mating between sterile males and wild females [29], which results in unviable eggs and the subsequent reduction of the target populations over successive generations. Because the SIT is insecticide-free and species-specific, it is considered an environment-friendly or neutral method, which has led to its increased implementation worldwide [30] following one of four possible strategies, namely eradication, suppression, containment, or prevention [31]. The unquestionable historical and current success of integrating the SIT in area-wide programs against many agricultural pest species [32,33] resulted in renewed interest for research and development and feasibility testing of the SIT against human disease vectors. In the 1970s, first attempts of using the SIT were successfully implemented against the malaria mosquitoes *Anopheles quadrimaculatus* and *Anopheles albimanus* [34,35]. Since then, scientific and technical advances, including increased knowledge on mosquito ecology and behavior, have improved prospects for the development and application of mosquito SIT strategies [36]. Various small-scale trials have been carried out to experimentally assess the feasibility of the SIT for malaria or arboviral vector control, notably in many countries in Europe, Southeast Asia, Latin America, and sub-Saharan Africa. For example, releases of sterile males significantly reduced the fertility of an *Ae. albopictus* population in small-scale pilot trials in Italy [37,38], Greece (A. Michaelakis, pers. Comm.) and China [39]. Most progress has so far been made in countries with relatively well-established research capacities, and through the close collaboration with and technical assistance of the Joint FAO/IAEA Programme of Nuclear Techniques in Food and Agriculture.

However, demonstrating the feasibility of SIT is not simply about corroborating an existing technology by integrating entomological knowledge and assessing the efficacy of sterile male releases in small pilot trials. Instead, developing a practical SIT program for mosquito control, for example in urban areas, should proceed along a number of specific steps [40,41], before the adoption and decision-making for area-wide application of the sterile male technology. Indeed, besides increasing scientific understanding of the vector ecology, establishing the technical capabilities and optimizing the production of sterile males, as well as elaborating strategies about how the sterile male releases can be calibrated, the SIT development pathway also presents other significant challenges including the social and regulatory validation of the usefulness or value of sterile males outside the laboratory.

Herein, we present an illustration of a phased conditional approach, which entails that the progression to next phase of testing is conditional on fulfilling all activities in the previous phase [40]. In this regard, we review a multi-year research and development process toward an SIT feasibility study for the control of *Ae. albopictus* on Reunion Island, which include scientific, technological, and experimental steps, as well as necessary social and regulatory components.

## 2. Opportunities for Establishing a Viable SIT Strategy

While recognizing the limitations of the current vector control strategy that combines targeted insecticide sprays with other prevention methods such as elimination of breeding sites and individual protection measures [42], there has been strong social and political interest to find alternative or complementary control options for *Ae. albopictus* by incorporating the SIT in the existing program. The integrated use of SIT against mosquitoes will most likely have greater potential in urban areas where the use of insecticide spraying is the subject of increasing criticisms. In response to this need, the French National Research Institute for Sustainable Development (IRD) initiated a scientific effort in 2009 entitled “Feasibility of Sterile Insect Technique for the control of *Aedes albopictus*, vectors of dengue and chikungunya viruses in Reunion Island,” with the goal to investigate the scientific and technological feasibility of the SIT against a social, economic, political, and regulatory background.

Why SIT testing on Reunion Island? There are several reasons to undertake SIT programs against disease vectors on Reunion Island. A first concern relates to public health: the established vector control strategy as described in [42], typically applied without prior ecological knowledge, seems ineffective in preventing the re-emergence of diseases such as chikungunya and dengue. The second concern relates to the continued application of insecticides and its negative impact on the environment and human health. As a result, it appears essential to explore the feasibility of using SIT as a complement to the existing vector control methods. This approach is well suited to help control mosquitoes and prevent outbreaks of vector-borne diseases in environmentally sensitive areas such as islands, while meeting the increasing public demand for vector control options that are safe for and protect human health. In addition, because of its geographical isolation, Reunion Island presents an ideal experimental location for the application of SIT in view that re-invasion of mosquitoes is less likely and easier to manage. Previous efficient use of SIT against invasive *Ae. albopictus* in other studies [37,38] has shown that it can be successfully implemented against this species, and with the necessary public acceptance. Regardless of these considerations, the final decision on whether and under what conditions to develop a framework for examining the feasibility of SIT in Reunion Island, as described herein, obviously benefited from prior political will and support (as detailed below).

Although there are twelve species of mosquitoes present on Reunion Island [7], *Ae. albopictus* is the most important vector of chikungunya and dengue. It is the only *Aedes* species thriving in urban areas on Reunion Island, with some rare exceptions on the west and south coast where *Ae. aegypti* also occurs in natural habitats surrounding the cities [7,11]. Since the chikungunya outbreak in 2005–2006, the organizational features of the local vector control agency have been greatly reinforced. It now has a competent workforce with 150 permanent vector control agents (compared to forty-five in June 2006), and sustainable financing to deliver reactive and programmatic interventions including entomological surveillance and epidemiological monitoring, integrated vector control, and community mobilization. This is very valuable, as the transition from a pilot study to a large-scale operational SIT program can be sustained and the impact maximized with optimal utilization of available resources.

## 3. Strategic Objectives Proposed for Establishing the SIT Feasibility Program in Reunion Island

**Figure 1:**
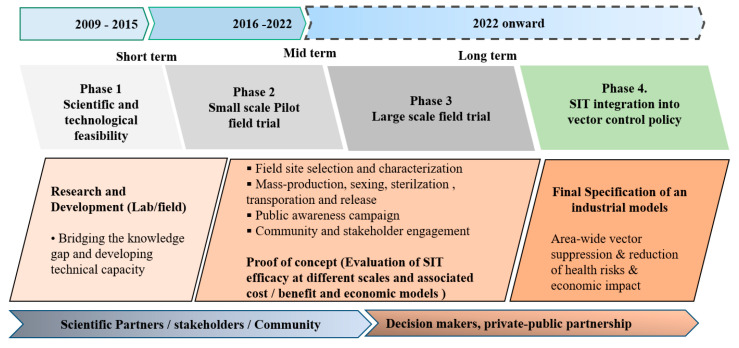
Strategic plan for developing and applying the sterile insect technique (SIT) against *Aedes albopictus* on Reunion Island. Legend. This figure summarizes the short, medium, and long-term SIT development plans. The strategic framework contains the necessary steps that should be taken to determine the overall strength of evidence (establish lines of evidence and knowledge gaps) needed when developing or applying SIT approaches for vector control [41]. The proposed strategy has been a continuous process of step-wise decision-making in response to scientific progress and technological development. The planned strategic option of SIT application against *Ae. albopictus* in Reunion Island focuses on suppression, rather than eradication [31], which is an almost unattainable goal.

The strategic plan was based on a phased conditional approach comprising four phases with short, medium, and long-term objectives (Figure 1): the initial phase 1 (completed) was carried out in 2009–2014 to determine whether the SIT is scientifically and technically an option for vector control on Reunion Island, focusing on *Ae. albopictus* but remaining alert to other mosquito vector species that may be potential targets of the SIT. The specific objective of this feasibility phase was to identify high-priority research opportunities to better understand the ecology and biology of the vector, and to address scientific and technological needs to undertake a field trial demonstrating the applicability and efficacy of the SIT. Phase 2 (ongoing) was built on the initial phase and aimed to design the intervention strategy and to implement a pilot trial emphasizing an integrated vector control approach. The efficacy and efficiency of the SIT will be assessed through relevant (1) entomological, (2) epidemiological, (3) environmental, and (4) socio-economic indicators. Phase 3 (planned) will focus on a long-term vision to technology transfer and establish the necessary infrastructures for the mass-production of mosquitoes and associated activities and procedures such as sterilization, packaging, transport, releases, monitoring etc. This phase would be supported by scientific partners, to create conditions for large-scale interventions, building on the success of previous (phase 2) activities. The phase 4 (foreseen) stresses the importance of political adoption with the integration of SIT into policies as a component of prevention strategies against vector-borne diseases transmission.

## 4. Challenges in the Development and Pilot Testing of SIT on Reunion Island—10 Years of Research and Development

Developing the SIT program from scratch has been a challenging process. In the beginning (2009–2010), there were still a number of knowledge gaps with respect to limited information on the biology, genetic, and ecology of the target species, and the lack of technological capacity for mass-rearing, sexing, sterilization, and field-testing. Subsequent constraints were the initial lack of confidence of the general public in the SIT strategy or to challenging regulatory prerequisites, or the lack of an appropriate regulatory framework for authorizing the release of irradiated males. This created a need for proactive and collaborative engagement with stakeholders’ representatives to help ensure that efforts to fast-track SIT development were in place. Ultimately, these challenges facilitated the development of local capacity in understanding aspects of mosquito ecology and developing specific technological solutions for mosquito mass-production. In parallel, we were proactively mounting awareness of the local public about the ongoing research activities. Despite the initial misconception about the SIT, the national and regional authorities were interested in and willing to give this technology an opportunity, through funding it, as indicated below. As the regulatory aspects were not yet available within the structure of the government, we spent time in setting scientific insights that mediated arguments about how the SIT is calibrated and the criteria for success or failure. Although we were still at an early experimental stage, scientific advances in recent years laid the ground for building up a regulatory environment from the very beginning, before planning and undertaking a more realistic testing in the field.

The above-mentioned constraints resulted in a long and costly process from the necessary feasibility prerequisites to the field pilot trials (10 years). The program budget, encompassing grants awarded and in-kind contributions was estimated at €4.5 million to support staff salary (60%) and the implementation of the R&D activities (40%). Funds for the pilot program from 2009 to 2020 were mobilized through an unprecedented collaboration between the two key national and regional funding organizations: the French Ministry of Health, and the Regional Council of Reunion through the European Regional Development fund, with additionally in-kind contributions provided by the IRD. The sections that follow are based on primary research with an example of multi-partner coordination that is needed to develop a multidisciplinary research base crucial to support SIT development and progression toward its application on Reunion Island. Table 1 shows the specific research priorities under the ongoing SIT feasibility project for the control of local *Ae. albopictus* populations at least on a small scale in the island.

The first fundamental objectives in phase 1 was to refine the current knowledge regarding the ecology and biology, including genetics and behavior of *Ae. albopictus* on Reunion Island. To this end, we used two complementary trajectories, one of which was collection of pre-existing data from the literature; while baseline data were collected as part of extensive entomological studies describing the geographic distribution [7,8,11,14,43], the genetic structure and phylogeny of local populations [15,44,45] as well as baseline data on the ecological requirement, and behavioral characteristics of the target species [12,46]. Phase 2, with an emphasis on fundamental research, produced new knowledge on two main *Aedes* species in the selected pilot test and comparison site, which will be key in supporting the SIT application as a viable option for *Ae. albopictus* control in Reunion Island.

The second specific objective was to initiate a colony of the target species *Ae. albopictus* and expand it toward a sizeable mass-rearing system. Key gaps in knowledge of behavioral traits such as daily activity pattern, mating capacity, and mating compatibility that are particularly relevant for SIT [47,48,49,50,51] were filled. In phase 2, research initiatives focused on an evaluation of existing sex separation techniques, irradiation procedures, and dose-response studies to establish the sterilizing doses to produce fully sterile but competitive males. Subsequently, the fitness and performance indicators including sexual behavior and longevity of laboratory-reared versus radiation-sterilized males, as well as the mating performance, sexual competitiveness of sterile males when released in the field, and their capability to induce sterility in wild female mosquitoes were assessed [52,53,54].

The third specific objective included mathematical models and simulations to assist in the evaluation of the impact of SIT on the spatial and temporal distribution and dynamics of *Ae. albopictus* populations [55]. A similar objective was pursued in phase 2, to test different models regarding the release strategy and to predict the impact of releases of sterilized males on the dynamics of the target populations. The development of models based on biological data (fitness, competition, dispersal, gene flow, etc.,) of wild and sterile males should facilitate developing release strategies and defining key indicators of their efficiency, especially in terms of location, period, and number of insects required [56,57,58,59]. The final objective dealt with qualitative and quantitative sociological studies to better understand the public perception and attitude regarding risks linked to the presence of *Ae. albopictus* [60,61]. Results of the phase 1 study indicated (a) high expectations of the local population about new vector control methods, mainly as a consequence of high nuisance and a high perception of risk, (b) large household expenditures for mosquito personal protection (c) conventional control considered relatively ineffective, and (d) confidence in the progress of science to reduce the number of mosquitoes [60]. These insights translated into an effective communication strategy that was easily understandable by the beneficiaries. The principles of our strategy stated that an effective communication would be the one that works transparently to inform the public and that enhances public acceptance. In this way, the insights phase laid the ground for both institutional and public-centric communication approaches. To better understand the public experience and perception prior to SIT pilot testing (Phase 2), we engaged in a series of quantitative surveys with individual interviews.

Overall, the phased research program, carried out from 2009—until 2020, was intended to acquire Reunion’s experience in the technological development of SIT, and to bring biological, technological, and socio-economic arguments confirming the feasibility of the large-scale implementation of SIT locally.

## 5. Pre-Release Entomological Field Studies

Interest in clarifying biology and ecology of *Ae. albopictus* has always been the predominant research strategy (combining laboratory and field experiments) toward establishing SIT. This section does not attempt to cover all aspects of the ecology of *Ae. albopictus* on Reunion Island within the SIT feasibility plan, but it aims to highlight the understanding of ecology, mainly inkling the population genetics, population dynamics, and behavior of *Ae. albopictus*, our target species.

### 5.1. Field Site Selection and Characterization

The use of SIT in urban areas remains a highly desirable goal for the following reason: the human populations are concentrated in cities of the island where the density of *Ae. albopictus* per unit area can be high [46], and cities are mostly located in coastal areas. Urbanized settings (or quarters) that are separated by mountain ridges and deep ravines that extend from the mountain sections down to the coast characterize each city. With the exception of passive dispersal, these natural barriers result in the relative isolation of the mosquito populations in each of the cities. Although *Ae. albopictus* is the only vector species present in most cities, the presence of sympatric *Ae. albopictus* and *Ae. aegypti* [13,14] was carefully considered in selecting the suitable sites for SIT pilot testing, as the co-occurrence of both *Aedes* mosquito species in the pilot site would add a level of complexity to the SIT trial design. While it is possible to release simultaneously sterile males of the two species, the release of a single species was considered as the more cost-effective option.

Noteworthy, the reduced geographical range of *Ae. aegypti* in Reunion Island [13] aroused as a consequence of competitive interactions between *Ae. aegypti* and *Ae. albopictus* and other ecological factors, which might have triggered the observed reduction of *Ae. aegypti* populations [14]. Vector control campaigns during the anti-malaria campaign in the fifties might also have accelerated this process [11]. In Reunion Island nowadays, *Ae. aegypti* finds its niche in an isolated small natural refuge. It is rarely encountered in the human environment and is not known to be anthropophilic. Consequently, all the efforts to prevent dengue fever and possibly other arboviruses is focused on the widespread *Ae. albopictus*. Thus, the use of SIT for selectively controlling this species on a large scale can result in large health benefits.

The context of the current SIT program in Reunion Island to demonstrate the feasibility of small-scale suppression of *Ae. albopictus* benefits from the absence of *Ae. aegypti* in the selected pilot site (as described below). At least at the pilot level, there is *a priori* no reason to expect a negative impact on non-target organisms—overall, unexpected detrimental effects of sterile male releases to suppress an invasive species such as *Ae. albopictus* are unlikely [62]. However, the presence of *Ae. aegypti*, although currently restricted to a small natural refuge located 20 km southeast of the main city, raises legitimate concerns about the possibility of this competent arboviral vector species expanding its distribution range across the island and taking over the vacant niche following *Ae. albopictus* suppression. With these considerations in mind, we have also initiated another SIT project targeting *Ae. aegypti* in the main natural refuge where it thrives. The primary aim of this ERC funded project (led by researchers at the CIRAD—The French Agricultural Research Centre for International Development) is to develop and assess the effectiveness of combining SIT with pyriproxyfen (a juvenile hormone analogous) to boost the impact of SIT [63]. We wish to compare the evolutionary response of the local *Ae. aegypti* to these different control pressures. This will enable the development of research-based capacity to establish a reactive and gradual SIT-based intervention system for different levels of *Ae. albopictus* and *Ae. aegypti* populations in different areas of the island, thereby ensuring sustainable benefit to public health [64,65]. This experience will also benefit several other regions in the tropics where both species are often present.

**Figure 2:**
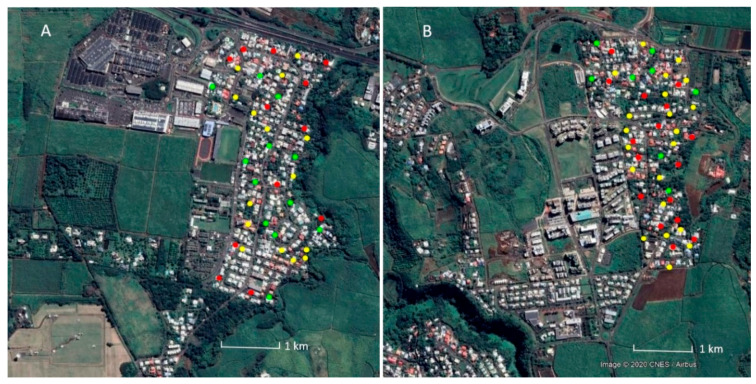
Aerial view of the two field sites: (**A**) Duparc and (**B**) Bois Rouge for SIT pilot testing in Reunion Island. Yellow and red circles indicate the positions of individual ovitraps and BG sentinel traps, respectively, while green circles show the parcels where both ovitraps and BG sentinel traps were deployed for *Aedes albopictus* population monitoring.

Limiting the focus on the ongoing research efforts to establish the feasibility of SIT for *Ae. albopictus* control, two similar, small, isolated urban areas were selected, i.e., Duparc as the treatment site and Bois-Rouge as the untreated control site. Both sites are ecologically representative of much of the urban settings of Reunion Island, and located on the north coast in the Sainte-Marie district (20°53′48″ S, 55°32′58″ E) (Figure 2), where *Ae. albopictus* is the only *Aedes* (*Stegomyia*) mosquito species present [8,12,15]. Duparc is located at an altitude of 50 to 80 m above sea level (asl) and covers a total surface area of ca. 42 ha. It is a residential area with 373 houses and isolated through natural topographic barriers, i.e., a deep ravine and shrubby area in the southeast, the expressway in the north connecting Saint-Denis to Saint Benoit, and sugarcane fields (width of 200 m) surrounding the residential area. Bois-Rouge is a residential zone of 24 ha situated 1.6 km southwest of Duparc, at an altitude of 150 to 250 m asl. This zone is surrounded by a large unoccupied green landscape, consisting of sugarcane fields of cane and market gardening. The two sites are separated by a 1000-m buffer of non-residential, agricultural land, mainly sugarcane plantations. Entomological surveys focused on the residential areas and the buffer, where feral *Ae. albopictus* populations are most likely to occur. From June to September, the relatively dry “austral winter” has temperatures between 16 and 25 °C on the coast. From December to April, temperatures of 18 to 33 °C are obtained on the coast during the summer rainy season that is also subject to the passage of several tropical depressions. Annual precipitation is on average 1500 ± 357 mm (data from 2011–2019) with the highest rainfall in December to March (www.meteofrance.re/, 2020).

### 5.2. Pre-Release Entomological Surveys

Entomological monitoring in the selected pilot sites was carried out over four consecutive years to determine the relative abundance, the reproductive capacity, and seasonal dynamics of the target species (Figure 1). The knowledge obtained through the pre-release surveys in 2013–2017 allowed the adaptation of the SIT pilot program to the specific characteristics of the release and control sites. The field monitoring was based on three complementary actions: (1) Ovitraps to collect baseline data on the temporal and spatial fluctuations in fecundity and egg viability; (2) BG traps to monitor the dynamics of the adult *Ae. albopictus* population; and (3) a series of mark-release-recapture (MRR) studies provided information on the population size, dispersal, and survival of fertile and laboratory-reared sterile *Ae. albopictus* males. Before the initiation of the surveys, each inhabitant living in the sites was informed about the project objectives and the entomological monitoring methods and permission obtained to deploy the traps. With the exception of a few cases, BG traps and ovitraps were placed in tandem in shaded areas of the backyards of residential parcels. Although compliance was high, a nearby parcel or shaded public area was selected as an alternative trap location in case residents did not grant permission. The paragraphs below summarize the status of the progress made with respect to the collection of entomological data.

### 5.3. Baseline Fecundity and Fertility in Wild Ae. albopictus Populations

A series of year-long entomological monitoring was carried out routinely from 2013 to 2018 in each pilot study site to assess the presence of container breeding mosquitoes and monitor the dynamics of *Ae. albopictus* fecundity (number of eggs laid) and fertility (egg hatch). Between 27 and 30 ovitraps and 20 to 25 BG traps were deployed in each study site (Figure 2). Eggs were collected in ovitraps once a week. Weekly and monthly egg abundance was used to establish the population dynamics of *Ae. albopictus* in the control and intervention site. Egg fertility is a useful parameter to evaluate the efficacy of control tactics such as SIT, and therefore, great emphasis was placed on standardizing egg-hatching methods. Egg viability (i.e., the proportion of eggs that hatched in each collected field sample) was monitored every two weeks.

In an earlier preliminary study [45], *Ae. albopictus* was the only mosquito species recorded in the samples collected in compatible containers and in the ovitraps. The results indicated a high (>80%) and constant mean ovitrap index (average proportion of ovitraps with eggs) in both Duparc and Bois-Rouge with the exception of August, when the index decreased to 50–70%. The results showed that *Ae. albopictus* mosquitoes breed throughout the year at both study sites. Average egg density in the ovitraps was high (>200 ± 45 eggs) especially from early October to mid-April. A maximum of 937 eggs were collected in a single ovitrap in the first week of December 2014. Egg counts fluctuated seasonally with lower egg density in the winter season from June to mid-October. The least productive months were August–September with less than 50 eggs/ovitrap. Such dramatically high trend in *Ae. albopictus* egg density per ovitrap has also been shown in Mauritius, where climatic conditions were found to significantly affect the ovitrap index [66]. A possible explanation for this effect is that temperature and/or rainfall patterns seemed to be reasonably effective predictors of time of peak mosquito abundance from November to May—cold weather and dryness being associated with low mosquito abundance in the winter. Egg hatch was high (>80%) with no seasonal trend recorded in both Duparc and Bois Rouge. Overall, the monitoring data over several years revealed low inter-annual variations in percentage of positive ovitraps and their productivity in eggs. However, the relatively high ovitrap productivity levels suggests that prior suppression of *Ae. albopictus* population through the elimination of potential breeding habitats, for example, will be required to maximize the effect of the sterile male release.

### 5.4. Seasonal Monitoring of Adult Ae. albopictus Population Density and Behavior

An efficient adult sampling method that can detect changes in the movement, distribution, density, and structure of the target wild population is a prerequisite to develop a well-planned intervention strategy. A commonly used sampling tool for wild adult *Aedes* populations is the BG sentinel trap, which uses a combination of olfactory and visual cues to attract individual mosquitoes. Preliminary studies on Reunion Island indicated that BG sentinel traps baited with commercial BG lure appeared to be less effective in trapping males than female mosquitoes. Female capture rates were always very low at 2–5 mosquitoes per trapping day [67]. Therefore, different attractants were tested in an attempt to enhance the efficiency of BG-sentinel traps for sampling wild males. Attractants such as BG lure, mouse, mice litter, mice odor, dry ice, CO_2_ alone or in combination with BG-lure were tested and compared for their efficacy in sampling *Ae. albopictus* females and males. Although BG traps baited with mice attracted significantly more *Ae*. *albopictus* males and females than other attractants [68], this was considered impractical for use in a large field monitoring program. In further studies, baiting the traps with CO_2_ plus lure sampled reasonable numbers of female and male *Ae. albopictus* [69], and this system was selected as the standard monitoring tool for the SIT program.

A series of mark-release-recapture (MRR) experiments were carried out to assess the size of wild populations and the dispersal of laboratory reared fertile and sterile males. These experiments were carried out in 2015, 2016, 2018, and 2019 in Duparc at regular intervals: in March–April (when the mosquito densities are relatively high), in June–July or in August–November (mosquito densities are at their lowest), and in November–December when mosquito densities are increasing. The MRR studies were carried out during different seasons to assess seasonal variations in adult population densities, survival, and dispersal. These data would facilitate decision-making regarding the appropriate time for releases, the required number and the quality of sterile males, frequencies of release, and the evaluation of the impact on sterile male releases on population abundance.

Wild mosquitoes are exposed to natural conditions, while mass-reared insects are exposed to fairly stable and controlled environmental conditions, which may cause some changes in the behavior of laboratory-reared males. To assess any potential difference in their behavior under field conditions, a series of MRR experiments were carried out to compare the dispersal and longevity of laboratory and field-collected *Ae. albopictus* male mosquitoes [70]. Each time, cohorts of approximately 2000–3000 males were marked with fluorescent powder, released and recaptured using BG sentinel traps for 14 consecutive days. The results indicate that the population density ranged from 650 to 750 wild males/ha and from 4800 to 6000 males/ha in the dry, cool season and the hot, rainy season, respectively. The reason for this variation in population size is unknown, but this is likely related to seasonal variability in temperature and rainfall [59,71,72]. This is consistent with our previous study on the classical *Stegomyia* indices (e.g., Breteau index, house index) on Reunion Island showing that the increase in *Ae. albopictus* densities coincide with increased rainfall and high temperature and humidity in the summer [43].

In most MRR experiments, dispersal and survival of released field-collected and laboratory-reared males were similar. Regardless of the season, 80–90% of the field-collected and laboratory-marked males were recaptured within 50 m from the release point and the recapture rate decreased for traps placed at further distances. The mean distances of the released field-collected males and laboratory males ranged from 46–55 m in the summer to 67–75 m in the winter. This observation is consistent with the generally accepted hypothesis that the active dispersal of *Ae. albopictus* males is generally assumed to be limited to 50–100 m [73,74]. During the summer, the survival probability of laboratory-reared males and field-collected males approximated 0.90 and 0.91 corresponding to an average life expectancy of 9.5 and 10.6 days, respectively. The daily survival probability of males in the winter was slightly lower, i.e., 0.88 (life expectancy of 7.8 days) for wild males, and 0.84 (life expectancy of 6.7 days) for laboratory males [70]. Both environmental factors [57,58,59] and climatic conditions at the time of the MRR experiments could have influenced the survival of the released males in the field. Some methods such as modeling and use of computers simulations are not discussed specifically herein, but have been addressed as part of the effort to understand the spatial and temporal dynamics of *Ae. albopictus*. A few mathematical models have been specifically developed and tested against the field observations described above, both to predict the environmental partitioning of mosquito behavior [57,58], and to simulate their population dynamics realistically [59]. These models also provided insight into environmental factors and weather-driven factors involved in *Ae. albopictus* populations and how these can be amenable to control using SIT interventions [55,72].

Based on the obtained data, the releases should start in July-September when the mosquito population size is the lowest. This will require producing and releasing a minimum of 6000 *Ae. albopictus* males/ha to achieve the previously predicted 10:1 sterile male: wild male ratio.

## 6. Colonization and Mass-Rearing of Local Strain of *Aedes albopictus*

In 2010, a small colony of *Ae. albopictus* was established from wild insects collected in St Denis. In an effort to streamline the rearing process, the rearing conditions were investigated in terms of photoperiod and temperature, larval diet, and blood-feeding regimes. The established colony showed a short larval development period (5–7 days at 28 °C and 80% RH), a high male mating success in laboratory cages (30 × 30 × 30 cm) and did not require initial selection for increased egg productivity. The main improvements took place in the larvae and adult rearing and environmental conditions, resulting to an initial production level of 5000–10,000 adult mosquitoes per week in the laboratory colony, which was sufficient to study key behavioral traits of the lab-reared insects [49,50,51,52,53].

Although the existing rearing procedures were not designed for mass-production, in the absence of an appropriate mass-rearing facility, the original rearing protocols [52,53,54] were adapted to increase the level of production using small laboratory cages (30 × 30 × 30 cm). In 2020, 58 generations of the *Ae. albopictus* strain have been reared with a stable production capacity of 50,000 males per week [75]. Productivity in the colony has been improved by calibrating the rearing parameters, the mosquito density and the male: female ratio per cage, female blood feeding system, and reproductive schedule including eggs collection and hatching, larval food, and feeding regimes. All these aspects have been extensively investigated to ensure optimal adult feeding and survival, synchrony in development and a high level of egg production [75]. It can be concluded that mass-rearing of *Ae. albopictus* on Reunion Island is possible at a relatively low cost—within limitations of the available space and personnel.

The current laboratory facility is, however, inadequate to produce sufficient *Ae. albopictus* males to achieve the required sterile to wild male ratios. The small size of the laboratory will not allow expanding the production level, and therefore, there is a need to progressively shift to a larger mass-rearing facility. The construction and equipping of a new mass-rearing facility is planned as part of a complementary project co-financed by the European Regional Development fund. We have recently completed a design of the facility with a predicted production potential of 200,000 to 250,000 sterile males per week. It includes four modular units incorporating all elements of the production from egg collection, larval rearing, sex separation, through irradiation and release. It is anticipated that the facility should be fully functional in July 2021, in time for the scheduled start of the pilot releases. Some of the important issues that will be addressed in the nearest future are the upscaling of the production level, environmental concerns, strain management, automation, sex separation, and irradiation under mass-rearing conditions, and standardized quality control methodology.

### 6.1. Sex Separation and Irradiation of Males of Laboratory-Reared Aedes albopictus

Establishing a SIT against disease-transmitting mosquitoes requires the separation of the sexes and the sterilization and release of male only mosquitoes. As soon as the laboratory colony of *Ae. albopictus* was established, a collaborative effort between the IAEA and the research group at IRD was set-up. After the transfer of new methods and equipment, these were tested and adapted to local conditions, including the larval rearing trays [76], mass-rearing cages [77], sexing techniques [78,79], irradiation processes, and quality control procedures [80,81]. Nearly all these aspects of sterile male production have been addressed during the period 2009–2016, as briefly presented below.

Releasing only males requires the ability to separate large numbers of females from males during the mass-rearing process. The status of the efforts to develop an effective sex separation method for mosquito SIT has also been reviewed in [78,79]. There is sufficient sexual dimorphism in our *Ae. albopictus* strain to allow reliable sex separation. The difference in pupation time (protandry) of male and female pupae facilitates the separation of fast-developing males pupae from the larvae, after which late developing male and female pupae are mechanically separated based on pupal size using the sieving method. However, a manual check is a logical mandatory step to detect and remove any contaminating females. This sexing method gives 1–2% female contamination, which is considered acceptable in view of the low production level so far established. However, in view of the need to upscale our production, the sieving system will need to be optimized. This is imperative so that even if a few irradiated females are released, there will not be an accrued risk of exposure to bites. There are, however, specific regulatory considerations that require avoidance of the release of sterile female mosquitoes during the SIT pilot testing in La Reunion [82].

Ionizing radiation exposure is used to induce sterility in mass reared flies for release in SIT program [83,84]. To ensure that released males are effective at inducing sterility in wild females, it is important that radiation procedures achieve an adequate level of sterility. The Reunion Island SIT pilot program currently uses the blood sterilization facility of the *Etablissement Français du Sang* located at the University Hospital Center in Saint-Denis, Reunion Island. The calibration of the X-ray irradiator (Blood X-RAD 13–19, Cegelec, France) and measurement of the dose rate (established 7 Gy. min^−1^ for the mosquito program) and the dosimetry accuracy were monitored every 6 months.

**Figure 3:**
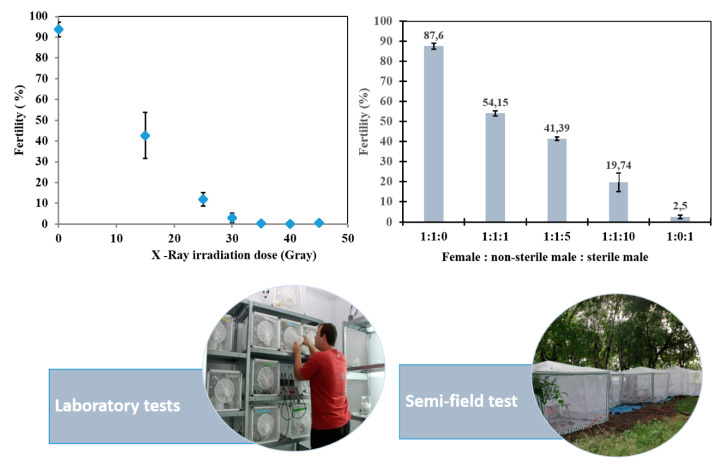
Dose-response curves of the sterility and mating competitiveness of sterile *Aedes albopictus* males from laboratory reared colony. Legend. The dose-sterility tests (unpublished data) were performed in laboratory cages while the mating competitiveness experiments were conducted in field cages. In all studies, mass-reared males were sterilized by X-ray radiation at 35Gy. Equal numbers of wild males, wild females, and mass-reared males were used in dose-sterility tests, whereas the competitiveness assays [54] tested the relationship between varying ratios of sterile to wild males and egg sterility (Re-drawn from [54]).

We have evaluated the effect of irradiation doses ranging from 0 to 50 Gy with the Blood X-RAD on the reproduction and longevity of the emerging *Ae. albopictus* males. Key factors that can influence sterility levels and quality of sterilized insects are: (1) The age at which pupae are irradiated (recommended pupae aged between 24 and 28 h); (2) the number of pupae per batch (1000–2000 pupae); (3) the oxygen concentration (pupae are essentially irradiated under hypoxia conditions). It is worth noting that *Ae. albopictus* males from different regions may exhibit different sensitivity to radiation, most likely due to genetic differences. This pattern is reflected in different sterility levels in males exposed to the same range of irradiation doses [85]. Figure 3 depicts the dose-sterility curves where radiation dose is plotted against the percentage egg hatch as a measure of sterility. The induced sterility in the female mosquitoes approached 98–99% at a dose of 35 to 40 Gy for our *Ae. albopictus* strain. This sterility level has been suggested as sufficient for the application of the SIT for *Ae. albopictus* [37,85]. Using the knowledge gained from previous small-scale studies, ongoing work focused on optimizing the irradiation of densely packed pupae per batch to ensure dose homogeneity. We have observed that irradiation of a batch of densely packed pupae (e.g., approx. 16,000 pupae placed in a column of 8 petri dishes, i.e., 2000 pupae per petri dish), resulted in 98.5% sterility levels in adult males. Removing the 1.5% residual fertility will require a higher irradiation dose, but this may result in compromised male-mating competitiveness [85,86]. It should be noted that the current consensus is that the highest possible sterility level should be obtained [87] and sterile males should exhibit a competitiveness that is comparable to that of fertile wild males.

### 6.2. Effect of Irradiation on Ae. albopictus Male Quality

An experiment was conducted under laboratory and semi-field conditions to understand the effect of radiation at the doses applied to *Ae. albopictus* pupae. Usually, batches of 500–1000 pupae aged 26–28 h (20–24 before adults emerge) placed in a Petri dish were exposed to X-ray radiation for 5 min at a dose rate of 7 Gy/min. Pupae treated with this radiation dose had an emergence rate of 97–98% after 24 hrs or more under laboratory conditions and <2% mortality of adult males within 2–4 days after emergence. We used standard mating tests in laboratory cages to compare behavioral and reproductive traits, such as sexual maturation, pre-copulation latency time, copula duration, mating success, multiple mating potential, time between matings, etc., [52]. *Aedes albopictus* males irradiated with 35 Gy were able to successfully mate with females, generally forming mating pairs within 30–45 s, similarly to untreated males. We consistently found no effect of irradiation treatment on most of these behavioral traits, but there was evidence that after five successful inseminations, sperm-depleted sterile males were unable to restore their sperm complement, compared to non-irradiated males that were able to replenish their sperm reserves following multiple mating opportunities [52,88]. The probability to inseminate females was also negatively correlated with the age of the sterile males, with irradiated males aged 5–10 days inseminating fewer females than untreated males. Importantly, this first study showed that *Ae. albopictus* sterile males, similarly to wild males, were able to inseminate an already mated female, as long as the mating occurred shortly (approximately 45–60 min) after the first mating [52].

Other biological parameters critical for the SIT such as daily flight pattern, flight performance [89], mating compatibility, and mating competitiveness [54,90,91] were tested both in the laboratory and in field-cages under natural conditions. Laboratory-reared and irradiated males had similar survival rates as compared with wild males, when placed in 60 × 60 × 60 cm^3^ cages outdoors [53,91]. Our observations are consistent with those of previously reported studies in Italy [37,38,84] and Mauritius [92], which showed that exposure of *Ae. albopictus* male pupae to radiation dose of 35–40 Gy does not significantly affect life history traits of emerging adults when compared to non-irradiated males. The survival in field cages of both types of males was drastically shorter in the presence of females. Mating competitiveness was assessed as the proportion of successful copulations obtained by differently aged sterile males with fertile females in sterile to untreated male ratios of 1:1, 5:1, 10:1. Laboratory mating competitiveness trials demonstrated that irradiated *Ae. albopictus* males aged 1–5 days, were able to mate successfully with females when in competition with untreated males derived either from the laboratory or from a field population [90]. As expected, 60–87% sterility was induced in eggs laid by females when sterile males were released at a ratio 5- to 10-fold relative to untreated non-treated males. Further experiments were carried out in large field cages that simulate better open field conditions. In preliminary competitiveness experiments in large field cages (L:3 m × W:3 m × H:2.5 m) placed under semi-field conditions, the competitiveness index of sterile males was 0.53 when sterile males were released after a 5-day period in laboratory conditions compared to only 0.14 when the release of sterile males occurred at 1-day following emergence [90]. In investigating the effects of male age at first mating as well as different release ratios, Damiens et al. [54] found that the mating competitiveness in field cages of 3-day old sterile males was significantly greater than when they were younger (1-day after emergence) or older (5-day-old). When 3-day old sterile males were in competition with untreated males at a 5:1 or 10:1 ratio, 60 and 87% sterility was induced in the female population, respectively. Releasing 3-day old sterile males appears to be the best option, but this requires maintaining the mosquitoes for 2 days in the facility and may entail additional operational costs in terms of diet, space, and staff.

### 6.3. Assessing the Field Behavior and Mating Capacity of Sterile Ae. albopictus Males

The first MRR experiments assessed survival and field dispersal of wild vs. laboratory-reared fertile *Ae. albopictus* males and provided a good basis for further experiments to assess field performance of sterile males. Sterile males need to have adequate survival and dispersal, as well as the ability to find and successfully mate with wild females. The performance of *Ae. albopictus* sterile males was assessed in three MRR experiments carried out in Duparc in June, September, and November 2019. On each occasion, 3000 wild males derived from field-collected eggs and the same number of laboratory-reared and irradiated males (exposed as pupae to 35 Gy of X rays) were differently marked with fluorescent dust and simultaneously released in the field (as described in [70]). A network of 20 BG traps baited with lure and CO_2_ was deployed on concentric annuli at 50, 100, 150, and 200 m from the release point and monitored every day over a 14-d period (C. Brengues et al. unpublished manuscript). Overall, the recapture rate of the wild and sterile males ranged from 6.5 to 7.4% and from 8.2 to 9.7%, respectively. In all cases/seasons, survival and dispersal of the wild and sterile males were comparable [70]. The estimated life span of wild and sterile males was 6.3 to 13.7 days and 5.3 to 10.6 days, respectively. This result indicates that the pilot suppression program should consider 1–2 release(s) per week. The average dispersal of the sterile and the wild males was comparable, with a significant seasonal variation (sterile vs. wild males: 42 m vs. 36 m in June, 65 vs. 59 m in September, 79 vs. 64 m in November, respectively). We have previously found that there were small but statistically significant temperature and/or rainfall variations during the dry season in comparison to wet season [59,71]. Congruent with earlier studies on non-sterile laboratory-reared males *Ae. albopictus* [67,70], this observation suggests that fluctuations in climatic conditions at the time of experiments could probably explain the observed differences in the dispersal of the released males across seasons. Regardless of the season, however, the results imply that achieving adequate coverage can be obtained when release points should be spaced 100 m or less.

A separate group of 3000 sterile males, marked with Rhodamine-B were added to the same MRR experiments and released at the same time as the two other groups. Results from laboratory studies have shown that this method is suitable to reliably mark *Ae. albopictus* males [93], and to detect females that have been inseminated by sterile males for up to 2 weeks (Damiens et al., unpublished data). Rhodamine marking has a number of advantages over fluorescent dust in that it is very practical and easy to apply through mixing with the sugar meal of the males [93]. Marked recaptured males can be easily detected with a fluorescent microscope. Wild collected females were dissected in the laboratory to assess presence of Rhodamine as an indicator of mating with marked sterile males. Although the number of sterile males that were released represented only 6% of the wild male population, 15% of the trapped wild females (Damiens et al. unpublished data) showed evidence of mating with a sterile males (presence of Rhodamine), indicating that the released sterile males were able to locate and mate with wild females. However, the level of induced sterility in the female population and its effect on overall population dynamics could not be assessed meaningfully. The most important conclusion of the experimental releases in the pilot site was that X-ray sterilized *Ae. albopictus* males showed good survival and behaved like wild males.

## 7. Communication, Social, and Regulatory Challenges for SIT Testing in Reunion Island

Despite demonstrating the technical feasibility of the SIT against *Ae. albopictus,* the transition from laboratory experiments to field trials, particularly in urban areas, was challenged by the public opinion and the local authorities. A better understanding of the public perception, concerns, and main misconceptions about SIT was obtained during earlier surveys. A significant part of the social survey examined the population-based survey data to identify key factors underlying the public acceptance or rejection of the use of SIT for mosquito control in Reunion Island [60,61]. This served as a basis to design a global communication strategy to inform the general public and enhance their understanding, as well as a mechanism for promoting active support and participation of all the relevant stakeholders involved, including public health agencies, local authorities, and government departments.

As a first step, an institutional communication campaign was developed and implemented to ensure that all stakeholders, including government departments and agencies, relevant local authorities and influential opinion leaders, had the information needed for decision-making during the planning and implementation phase. Second, a public awareness plan was developed to inform and enhance public understanding, as well as to address public controversies and gaining public trust. This phased communication and social mobilization strategy was implemented with the assistance of local media, the development and dissemination of brochures, as well as the development of a website (www.tis.re). We relied on external communication experts selected via a public tender procedure, to advise us on the actions necessary to achieve our public awareness campaign throughout the project. This solution was preferred because such intervention exceeds the field of competence of members of our scientific team; it also ensures designing more objective and easy-to-read non-technical dissemination content.

Transparency and positive response to requests for interviews from journalists enhanced media relations throughout the different phases of the project. This relationship was based on two overlapping pillars: the first aimed at controlling the communication schedule to the media (coverage of field operations, interim or final reports, press conferences during project meetings, etc.,) following the progress of the project step-by-step, and was planned within the well-established institutional (IRD) communication frameworks. The second approach, beyond the scheduled communication plan, was less predictable and depended on ad hoc demand from the media for information. These requests were addressed by well-informed spoke-persons, usually the scientific leaders of the project, to ensure consistency over time and maintain legitimacy. Over the past 5 years, major local media such as newspapers, radio and television channels, and their related online platforms on average covered 2–3 SIT related information a month. The media coverage focused mainly on topics of greatest public interest or concern, such as the circulation of dengue virus, but it also provided a constant chronicle of the state of scientific advances and the potential solution the development of SIT could bring to the health problems related to *Ae. albopictus* in Reunion Island. The level of media coverage suggests that public interest in the SIT has been constant and allowed gaining public confidence. The recent perception surveys among residents in La Reunion, demonstrated that repeated information was likely to increase social acceptance for SIT (see also www.tis.re).

Even though the development of SIT in La Reunion could offer a safer and effective tool to control and prevent a range of mosquito-borne diseases, its large-scale implementation depends ultimately on how people perceive this technology. In the past decades, several biotechnological or medical innovations (like new vaccines) have faced skepticism and sometimes opposition from large segments of the general public in both developing and developed countries. To assess whether the use of the SIT on Reunion island might be prone to social rejection, a large face-to-face survey was carried out in October 2018 among a representative sample of the adult population (n > 1000) (Raude et al., unpublished data). The study was based on an international questionnaire developed in Europe in the late nineties to investigate public perceptions and attitudes related to sensitive technologies and applications from the life sciences [94,95]. Respondents were asked whether they thought each of the six technologies—natural plant-based repellent, mosquito traps, indoor residual spraying, GM organisms, sterile insect technique, and outdoor space spraying—was effective to control mosquitoes, risky for human health or the environment, fundamentally unnatural, and to be encouraged in Reunion.

**Figure 4:**
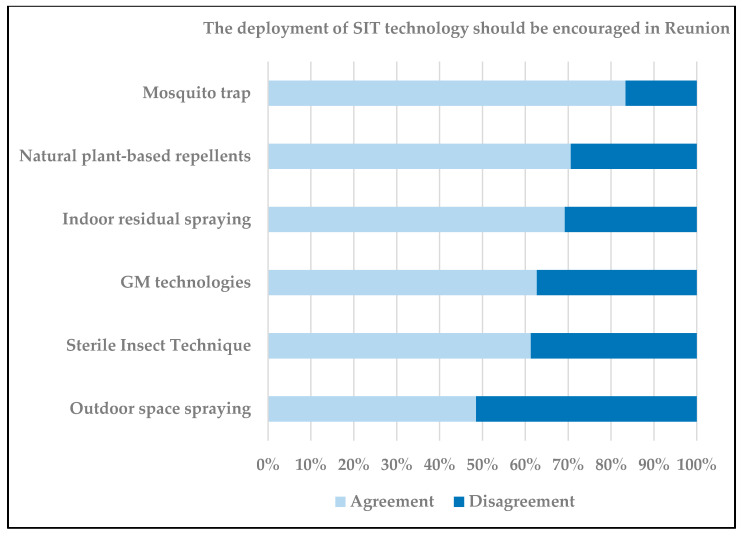
Index of overall social support of different mosquito control technologies.

The rates of public encouragement, which represents an index of overall support for each application, are presented on a percentage scale in Figure 4. With the exception of the chemical insecticides, a large majority of the respondents expressed support to all these mosquito control technologies. Although the SIT is still a relatively unfamiliar topic in Reunion, this data show that there was until recently no local hostility to the development of this technique. A multiple regression analysis, in which encouragement was regressed on the effectiveness, naturalness, and riskiness variables indicated that two independent variables—effectiveness and riskiness for human health—were the best predictors of the support for SIT. The combination of these predictors allowed explaining about 50% of the variance in encouragement and providing 80% of good prediction. This suggests that the structure of judgment and attitudes related to mosquito control techniques can be modelled through a simple and frugal decision-making framework based on the risk and benefit perceived by the public.

What other challenges remain for sterile male release in Reunion Island? So far effort to establish the SIT feasibility program for *Ae. albopictus* control on the island—through the process of setting up a requisite scientific and technological capacity—has been driven by researchers. Two other forces are of considerable importance, including the need to consider prior public acceptance of the proposed intervention (see above) coupled with the approval of the local public authorities. The local authority’s role here is extensive through its involvement in research planning, funding, and authorization of the delivery of SIT into the field. Upstream of planning the pilot phase, we put out a call to the local authorities: do we require formal approval for sterile male release in the field? The local authority needed to clarify its regulatory responsibilities and the legislative framework under which the open-field release of sterile males should be authorized. It is in this context that the French Ministry of Health and the Ministry of Ecological Transitions have jointly commissioned the French Agency of Biodiversity (AFB), as well as the High Council of Public Health and an ad hoc advisory panel with experts from various government agencies and universities to examine the current and potential future role of SIT-based technologies for vector control in Reunion Island. Through this process, we experienced a significant delay (almost 5 years) before securing official authorization for experimental releases of radiation-sterilized *Ae. albopictus* males. The delay was mainly due to: (i) The lack of general legislation in place in relation to SIT application for vector control in France and more generally in Europe; (ii) the uncertainty regarding potential speculative risks to human health from SIT [82]; and (iii) uncertainties about detrimental ecological or non-target effects (on mosquito predators, substitution by other mosquito species) [96]. As mentioned above, communication about the advances in SIT research and proactive consultation with key stakeholders have increased awareness and understanding, and allowed the authorities to identify solutions to the overall decision-making process. After a parallel oversight from the Departmental Council for the Environmental, Health and Technological Risks (CODERST) at convened full board review meetings, a major breakthrough for achieving this goal has been the prefectural decree published in June 2019 to authorize the field release of radiation-sterilized males in Reunion Island (http://www.reunion.gouv.fr/autorisation-du-1er-lacher-de-moustiques-steriles-a5365.html). This has enabled the first MRR using sterile *Ae. albopictus* males in 2019 and the pilot suppression trial is expected to start in 2021.

## 8. Conclusions and Perspectives

The multi-scale research strategy that incorporates laboratory, semi-field and field experiments have allowed gathering a large volume of scientific data to assess the feasibility of SIT for *Aedes* mosquito suppression in Reunion Island. Pre-release field research activities ensured the collection of data on the biology, ecology, and dynamics of the target population. These have been of direct benefit to the planning and rational implementation of the pilot SIT trial, and provided supplemental information to current vector control operations on Reunion Island. Pro-active engagement of the vector control agency throughout the field studies was therefore of paramount importance. On the same basis, we have developed the necessary capacity to become familiar with all the stages of the production, sexing, sterilization combining improved experimental protocols to assess the quality of sterile males. All the stages of field releases to evaluate the performance of sterile males have been successfully carried out, including the marking (either with fluorescent powder or with rhodamine-B) of irradiated males, their recapture and estimating their survival and dispersal capacity. The finding that they are able to mate with wild females argues well for the future pilot tests. However, research is still needed to demonstrate the potential of sterile male releases—particularly through small-scale field trials—to achieve a medium and long-term reduction in egg fertility and changes in the adult density of the target population and risk of disease transmission. We acknowledge the political and economic pressures on an ongoing SIT program but the demonstration of the SIT effectiveness could save critical resources and provide useful information for the next step, thereby increasing the chance for future success.

The current plan aims at establishing a mass-production facility for increasing the production level to 200,000 to 250,000 sterile males per week to meet the program requirements. With this challenge in mind, and considering the need for competitive sterile insects, [29,97], the focus will be on quality rather than quantity. History has shown that the less-successful SIT programs have been constrained by a low competitiveness of the sterile males [29,33]. Seasonal variation in population density has been demonstrated and this information will be used to target the population at its most vulnerable time [70]. Therefore, releases will begin in the dry winter period of June–September and early summer. Given that our target areas is relatively small and easily accessible, using aerial releases [98] will not be cost-effective. Therefore, the program will rely on ground releases of a minimum of 6000 sterile males per hectare targeting to be 10-times the estimated size of the wild males’ population in the winter. Sterile males will be released from predetermined release points separated by no more than 100 m. The frequency at which sterile males can be released is limited by the production level in the rearing facility. Within the constraints of the weekly production of the required sterile males, the pilot interventions will require the simultaneous use of different tools in an integrated vector management approach. The pilot release initially scheduled to start in June 2020 have been postponed, mostly because of the COVID-19 pandemic. New priorities under this project are expected to focus on workflow automation for mass-production of sterile males, pilot intervention with release of sterile males in conjunction with compatible vector control methods followed by an evaluation. Overall, this “holistic” program for establishing the feasibility of SIT as a component of *Ae. albopictus* control is being developed to address a real and urgent public health problem caused by *Ae. albopictus* and to meet a societal demand for a more targeted, environment-friendly and credible alternative to the use of insecticides for vector control strategy. The development of SIT in Reunion is therefore in line with political and public expectations.

## Figures and Tables

**Table 1:** Articulation of scientific research priorities in phases 1, 2, and 3 of the SIT program in Reunion Island.

R&D Priorities	Phase 1—Feasibility Studies (Filling the Knowledge Gap and Developing Technological Requisite)	Phase 2—Small Scale Pilot Tests Under Field Conditions (as Validated in Phase 1 Feasibility Studies)	Phase 3—Large-Scale Implementation (If Efficacy Is Proven in Pilot Test)
Biology and ecology of the target species	Refine knowledge on the ecology, biology, genetics, vector behavior, etc.,) of *Aedes albopictus* and *Aedes aegypti*	(1) Selection and characterization of pilot field sites based on entomological monitoring and studies on the behaviors of the target population. (2) Implementation of the SIT-based vector suppression in real conditions and efficacy testing. (3) Evaluation of SIT efficacy based on entomological, social, environmental and economic) indicators.	(1) Selection of the testing zones. (2) Implementation of the SIT-based vector suppression in real conditions and efficacy testing. (3) Evaluation of SIT efficacy based on entomological epidemiological, social, environmental and economic indicators. (4) Surveillance and monitoring.
Technological component (rearing, sexing, sterilization)	Establish the colony of the target species and improve capacity and knowledge on rearing, sexing, sterilization, and on key behavioral traits	Up scaling the mass rearing and (infrastructure construction, etc.,) and strengthening of the release strategy and quality control	Technology transfer, business model for transition to large (industrial) scale production of sterile males. Implementation tools (transport, release, etc.,).
Modeling and simulations	Modeling of the methodology and release of irradiated males (dispersion, dynamics, etc.,)	Models validation based on efficiency indicators (entomological, epidemiological, social, environmental and economic) parameters. Cost/efficacy analysis and model of integrated strategies	Confirm/validate the predictions of epidemiological models. Predictions to other impact indicators. Cost-benefit analysis associated with large-scale SIT application.
Communication and social sciences component	Refine knowledge (existing social perception, attitude and practices T0; survey of costs of existing control techniques), and social levers for the acceptability of the project. Inform the public	(1) Inform the general public and stakeholders to enhance their understanding and support. (2) Analysis of the evolution of social acceptance and engagement. (3) Cost/effectiveness analysis of combined vector control strategies. (4) Economy model for the transition to industrial production scale	Public awareness and influence of SIT-based vector control strategy on acceptability and behavior change.
